# Molecular interaction between three novel amino acid based deep eutectic solvents with surface active ionic liquid: A comparative study

**DOI:** 10.1016/j.heliyon.2024.e35598

**Published:** 2024-08-05

**Authors:** Manoj Kumar Banjare, Benvikram Barman, Kamalakanta Behera, Javed Masood Khan, Ramesh Kumar Banjare, Siddharth Pandey, Kallol Kumar Ghosh

**Affiliations:** a Department of Chemistry (MSS), MATS University, Pagaria Complex, Pandri, Raipur, Chhattisgarh, 492004, India; b Department of Chemistry, University of Allahabad, Prayagraj, Uttar Pradesh, 211002, India; c Department of Food Science and Nutrition, College of Food and Agricultural Sciences, King Saud University, Riyadh, 11451, Saudi Arabia; d Department of Chemistry(MSET), MATS University, Gullu Campus, Arang, Raipur, C.G., 493441, India; e Department of Chemistry, Indian Institute of Technology Delhi, Hauz Khas, New Delhi, 110016, India; f School of Studies in Chemistry, Pt. Ravishankar Shukla University, Raipur, Chhattisgarh, 492010, India

**Keywords:** 1-Decyl-3-methylimidazolium chloride, Amino acid-based deep eutectic solvents, CMC, Interfacial parameters, FTIR

## Abstract

Interaction between a surface active ionic liquid (IL) viz. 1-decyl-3-methylimidazolium chloride [Dmim][Cl] with three novel amino acid-based deep eutectic solvents (DES, consisting of choline chloride and l-methionine (DES1), l-phenylalanine (DES2), and l-glutamine (DES3) in a 1: 2 mol ratio) is studied. Several techniques, including surface tension, fluorescence, UV–visible spectroscopy, and Fourier transform infrared (FTIR), were used to investigate the key micellar properties and intermolecular interactions between the IL and DESs. All the DESs studied here facilitate the micellization process successfully lowering the critical micelle concentrations (CMC) of [Dmim][Cl] with addition of 5 wt% and 10 wt% of DESs. In decreasing order of DES2 > DES1 > DES3, the affinity to promote IL [Dmim][Cl] aggregation within aqueous DES solutions. Additionally, the CMC values as well as the surface tension at CMC are both noticeably reduced significantly by DES2. The surface tension method determines how three amino acid-based DESs affect the CMC, Г_max_, π_CMC_, A_min_ and pC_20_ of micellization. When IL [Dmim][Cl] forms micelles within DES solutions, the solvophobic effect predominates, and the intermolecular hydrogen-bond interaction helps to form micelles. FTIR was used to examine the molecular interactions and structural changes of the ionic liquid self-assemblies in aqueous DESs. The results show that the presence of DESs greatly aids in the micellization of [Dmim][Cl], and to a greater extent for DES2 than for DES1/DES3. The colloidal properties of DES and their mixtures are advantageous for the solubility, micellization, and other features of ionic liquids; further details on this positive observation are provided in the results and discussion. In the areas of micellization, CMC, synthesis, catalysis, and environmental, biological, and pharmaceutical applications, among others, DESs are extremely useful.

## Highlight

• Study the interaction of DmimCl with DES.
• The CMCs values are reduced with the addition of DES order is DES2>DES1>DES3.
• In increase in wt% of DES the spontaneity of aggregation of DmimCl is increased.
• Fluorescence and UV–visible methods shows the confirm CMC values for all three DmimCl micellar systems.
• Electrostatic, H- bonds, hydrophobic interactions, and van der Waal interactions were examined by FTIR.

## Graphical abstract

Interaction of IL 1-decyl-3-methylimidazolium chloride with deep eutectic solvents (DES).

**Figure:**
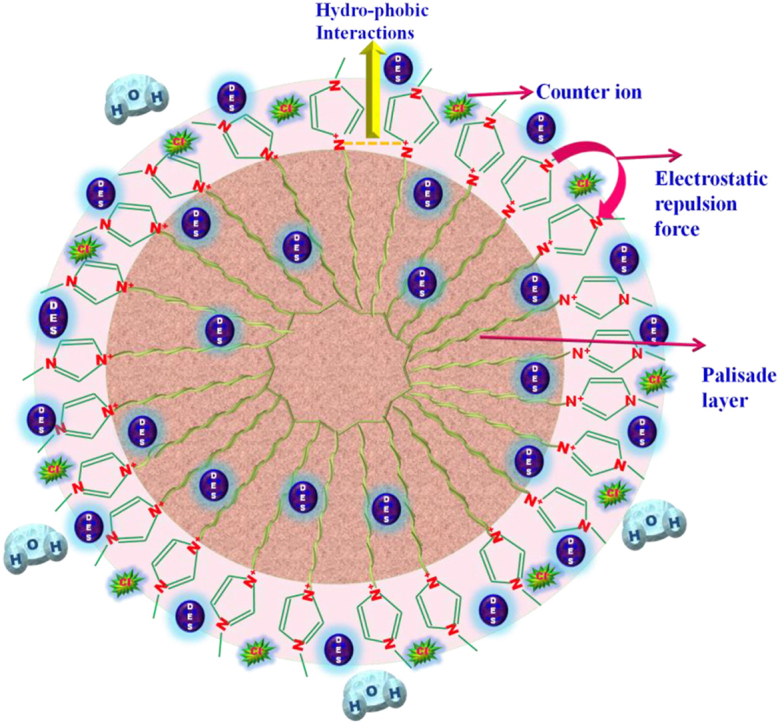

## 1 Introduction

Ionic liquids (ILs) have dominated the advancement of “green” or “sustainable” chemistry ever since the 12 principles of green chemistry were published in 1998 [1,2]. The late Ken Seddon's passionate support for ILs as environmentally friendly solvents [3,4] and Tom Welton's authoritative review article from 1999 both contributed to the enormous rise in popularity of these esoteric solvents [5]. ILs are solvents that only contain ions and no neutral molecules, therefore as a result of their shallow vapor pressure, they are almost non-volatile [6]. The fact that ILs are less volatile than typical molecular organic solvents, which are volatile organic compounds (VOCs), contributes to their “green” reputation as being safer and more environmentally friendly [7].

Long-chain ionic liquids (ILs), which are made up of hydrophilic ionic groups and hydrophobic organic branching chains, can self-assemble into a variety of structures [8]. One example of an amphiphilic compound is the long-chain imidazole IL, which resembles cationic surfactants in structure. A hydrophilic “head group” may be thought of as the positively charged imidazole ring, and a hydrophobic “tail group” could be thought of as the lengthy alkyl chain [9]. Since they can create micelles, vesicles, and other aggregates in aqueous solution, long-chain imidazole ILs have properties similar to those of surface-active agents [10]. Long-chain imidazole ILs have been found to have lower melting points, better solvent solubility, and marginally better surface activity than conventional ionic surfactants [11]. Long-chain IL self-assembly in aqueous solutions has so far been the subject of substantial research. It is quite intriguing from the standpoint of both scientific investigation and practical application to examine the self-assembly behavior of ILs in various solvents [12].

Eutectic mixtures known as deep eutectic solvents (DESs) have melting points that are significantly lower than those of the individual components. They are made up of two to three pieces that associate with one another thanks to hydrogen bonds [13]. The advantages of DESs over organic solvents are low volatility, little or no toxicity, a wide range of liquid usage temperatures, a wide range of electrochemical potential windows, biodegradability and low cost [14]. For usage in electrochemistry, separation techniques, organic synthesis, material synthesis, bio-transformation, and catalysis, DESs have been dubbed a new class of environmentally benign solvent and “soft” functional material [15].

The amphiphile assemblages in DESs are largely unknown, despite having a wide range of uses [16]. There have been numerous studies on the use of DESs as solvents for amphiphile assemblies, including one on the self-aggregation of sodium dodecyl sulfate, phosphatidylcholine lipids, and cationic surfactants from the n-alkyl trimethylammonium family in ChCl/gly and ChCl/urea [17]. On the subject of DESs and ionic liquids, a small number of studies have already been conducted. Knowing about DES synthesis, characterization, and characteristics is very beneficial.

Zhang et al. [18], used several methods, such as fluorescence, SAXS, and FTIR, to investigate the aggregation behavior of 1-alkyl-3-methylimidazolium chloride [CnmimCl] in a DES. When CnmimCl is micellized in DES, the solvophobic effect predominates, and intermolecular hydrogen bonds interact favorably to encourage micelle formation. The Cu_3_(BTC)_2_ was synthesized using micellar solutions. Cu_3_(BTC)_2_ nanocrystals with mesoporous architectures have been formed, according to XRD, SEM, and TEM. Ghosh et al. [19] used fluorescence, UV–vis, DLS, and FT-IR to examine the aggregation behavior of 1-butyl-3-methylimidazolium octyl sulfate [Bmim][OS] within aqueous DESs. We quantify the size and local microenvironment of the aggregates using steady-state fluorescence and DLS measurements, respectively. Given that the critical micelle concentration is substantially lower, and the aggregation number (N_agg_) is larger in DES solutions than in water, this suggests that the micellization process of the IL [Bmim][OS] is greatly preferred in DES solutions. FT-IR spectroscopy analysis reveals [Bmim][OS] molecules' molecular interactions in DESs. The antidepressant medication promazine hydrochloride (PH) was also studied using these methods to examine its interaction with IL.

**Scheme 1:**
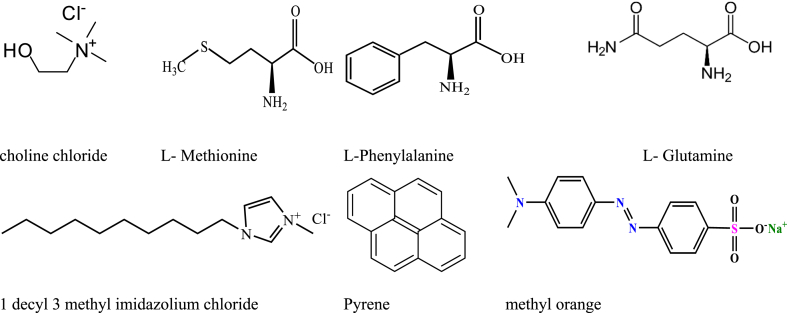
Molecular structure of choline chloride, l-methionine, l-phenylalanine, l-glutamine, 1-decyl-3- methylimidazolium chloride, pyrene, and methyl orange.

The work that is the subject of this paper has been divided into three sections. First, using choline chloride (ChCl) as the hydrogen bond acceptor and amino acids as donors (viz. l-methionine, l-phenylalanine, and l-glutamine) in a 1: 2-mol ratio, we created three DESs based on amino acids, which were then characterized by Fourier transform infrared (FTIR). Second, several micellar, interfacial parameters have been studied for the combined micellization of long-chain IL [Dmim][Cl] within 5 and 10 wt% of aqueous DESs solutions. Thirdly, we used FTIR to examine how IL [Dmim][Cl] interacts with DESs at the molecular level. The physiochemical characteristics of mixed micellar media have been investigated using a variety of techniques, including fluorescence, UV–visible spectroscopy, and surface tension. The chemical makeup of choline chloride, l-methionine, l-phenylalanine, and l-glutamine, as well as 1-decyl-3-methylimidazolium chloride, methyl orange and pyrene, is shown in Scheme 1.

## 2 Experimental section

### 2.1 Materials

The three purists-grade amino acids l-methionine (98.10 %), l-phenylalanine (98.28 %), and l-glutamine (97.10 %), as well as choline chloride (99.0 %), potassium bromide (99.0 %), and 1-decyl-3-methylimidazolium chloride (99.0 %), were bought from Sigma-Aldrich in Germany and utilized as-is. We used millipore water to create all of the aqueous solutions.

### 2.2 Methods

#### 2.2.1 Stalagmometer

The surface tension of various compounds including the chemical 1-decyl-3-methylimidazolium chloride and DESs is measured using stalagmometers (ABGIL Borosilicate, India). Before the start of these investigations, the stalagmometer was calibrated using double-distilled water and all of the apparatus was then cleaned and rinsed [20]. At that point, the surface tension method was used by the “Drop Volume Count” perspective to identify prepared solutions. The prepared solution was filled to just above its prescribed mark level and then allowed to flow freely to count the number of drops that passed through the capillary. To ensure accuracy, the droplet count was measured twice. By counting the droplets of the pure and solution stated above, the CMC and surface tension were computed.

#### 2.2.2 UV–visible spectrophotometer

The UV–vis absorbance was measured in a Varian Cary Eclipsed-60 UV–vis spectrophotometer using quartz cuvettes with a 10 mm path length. Between 200 and 600 nm in wavelength, the spectra were captured. The concentration of the DmimCl solution was raised from below CMC to above CMC using a Hamilton micro syringe and a concentrated solution was introduced progressively to water.

#### 2.2.3 Fluorescence

A fluorescence spectrophotometer from Agilent Technology that was connected to a computer was used to conduct steady-state fluorescence tests. The 1.0 nm excitation and emission slit widths were maintained. The sample cell was filled with the necessary volume of freshly made DmimCl/DESs solution containing the probe pyrene in a typical experiment. The DmimCl/DES solution had a pyrene concentration of 5 × 10^−3^ M.

#### 2.2.4 Viscosity

The binary mixtures and pure [Dmim][Cl] viscosities were measured using a capillary viscometer that was purchased from ABGIL Borosilicate, India. The viscosity was used to calibrate the viscometer constant after the capillary viscometer had been cleaned with acetone/methanol before the experiment. The viscometer was immersed in an oil bath while being filled with the solution under study and was being controlled by a thermostat with a ±0.01 K uncertainty. An electronic stopwatch with a 0.01 s margin of error was used to measure the sample's efflux time via the capillary. The viscosities were calculated using the efflux time multiplier and the viscometer constant [21]. Viscosity was measured in triplicate during the measurement, and the average was supplied. With a confidence level of 0.95 and accounting for sample contaminants, mixture preparation, and measurement technique, the relative extended uncertainty of viscosity in this study is stated as being less than 0.08. The relative viscosities were determined using Eq (1).

(1) $$\eta_{r}=\frac{\eta_{s}}{\eta_{0}}$$

where η_ο_ is the viscosity of the pure solvent, η_r_ is the relative viscosity of the system, and η_s_ is the viscosity of the solution.

#### 2.2.5 FTIR

On a Bruker Optic, Germany spectrophotometer, FT-IR spectra were captured using the KBr disk method. Samples were created as 100 mg KBr disks containing 1 mg complex. The FTIR measurements were carried out for DESs based on 1-decyl-3-methylimidazolium chloride and combinations of choline chloride and three amino acids (l-methionine, l-phenylalanine, and l-glutamine) in the scanning range of 4000–400 cm^−1^ at room temperature [22].

### 2.3 Synthesis of amino acid-based DESs

**Scheme 2:**
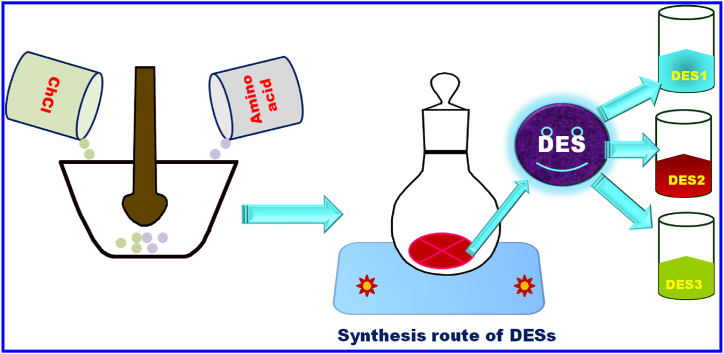
A Schematic representation for the synthesis of amino acid-based DESs [23].

To produce DESs, choline chloride was mixed in a 1:2 M ratio with the amino acids l-methionine, l-phenylalanine, and l-glutamine. The mixture was then heated at 80 °C for 4 h at air pressure while being stirred continuously shown in Scheme 2. The DESs were validated by the FT-IR spectra after the manufactured DESs were all dried for 24 h in a vacuum oven at 6 °C. Table 1 presents the results [[23], [24], [25]].

**Table 1:** Comparisons of IR spectra between the ChCl and DES- 1 (ChCl: l-methionine), DES- 2 (ChCl: l-phenylalanine), and DES- 3 (ChCl: l-glutamine). Error in data = 2 %.

Observed IR frequencies (cm^−1^)				
Assignments	ChCl	ChCl- l-methionine (DES1)	ChCl- l-phenylalanine (DES2)	ChCl-l-Glutamine (DES3)
Asymmetrical NH_2_ stretching			3207.09	3210.43
-OH (Alcohol) Stretching	3222.21	3395.20		–
-OH (carboxylic acid) Stretching			3027.45	
C–H (Alkane) stretching		2913.20		
N–H bending	1672.93, 1611.62	1714.71	1658.86	1652.21
C–H bending	1483.45	1480.95	1504.91, 1441.49	1573.69, 1403.95
-O-H (Carboxylic acid)		1410.20	–	–
NH bend + CN bend	1352.48	1398.67		
CH_2_ deformation	1149.07		1218.31	1290.88
C–C stretching + other vibrations	1090.55 1018.10			
N–C–C bending	954.01	983.41		
Wagging CO, wagging NH_2_ + CO out of phase	789.61		819.21	
C–H Strong, bending			749.81	
C=C (alkene) Benzene			695.82	

## 3 Results and discussion

### 3.1 Characterization of synthesized three amino acid-based DESs by FTIR method

**Scheme 3:**
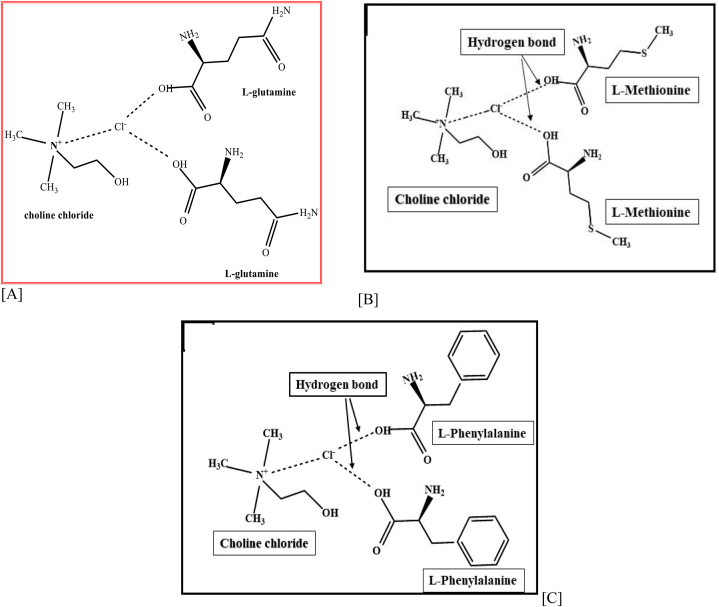
A molecular interaction representation for the synthesis of amino acids and choline chloride.

We have studied the interaction of hydrogen bond acceptor (HBA) viz. ChCl and hydrogen bond donor (HBD) amino acids such as l-methionine (DES1), l-phenylalanine (DES2), l-glutamine (DES3), and also the interaction between ChCl: l-methionine (DES1), ChCl: l-phenylalanine (DES2), and ChCl: l-glutamine (DES3) by using FT-IR spectroscopy [[26], [27], [28]]. It is useful for describing the H-bond interactions between them was shown in Scheme 3 [29].

**Fig. 1:**
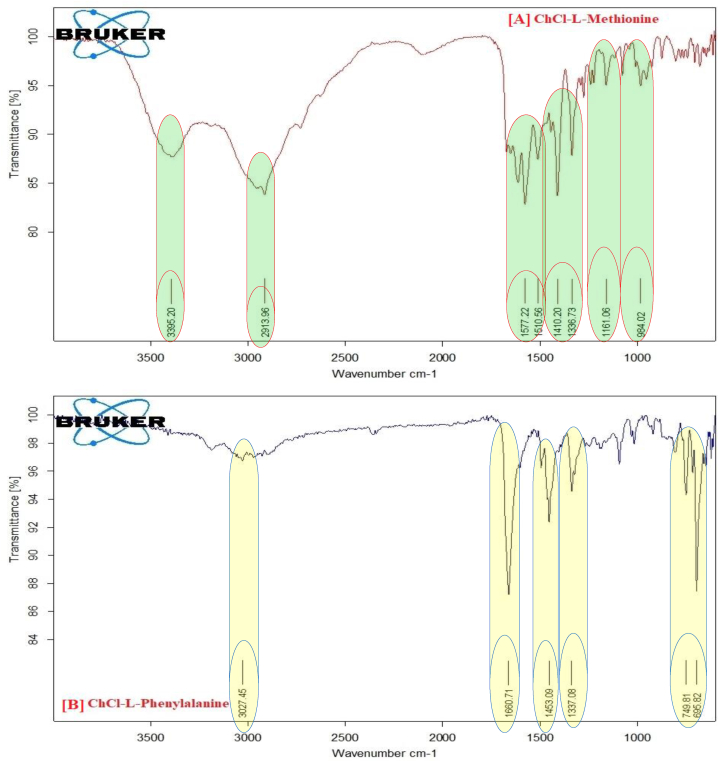

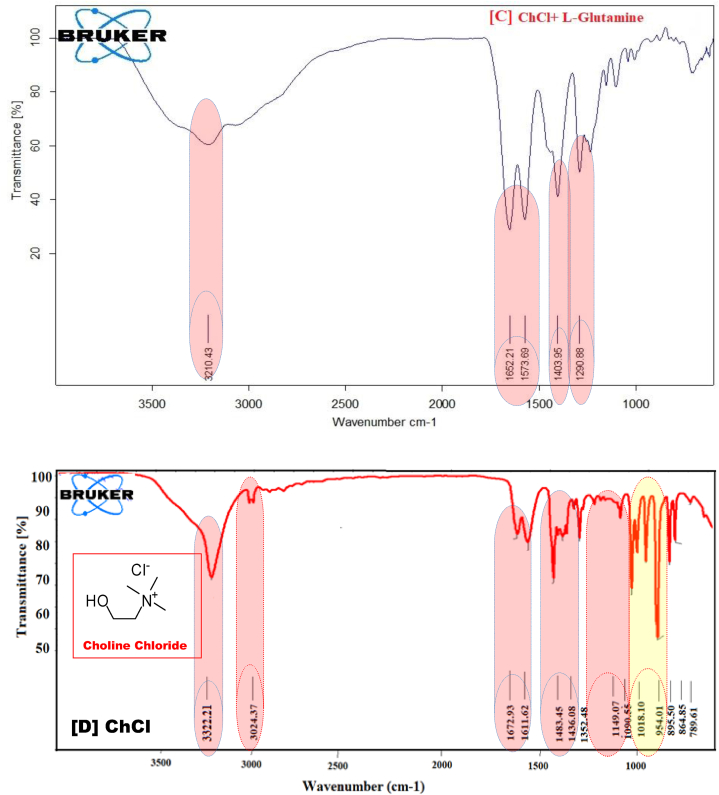
FT-IR spectra of synthesized amino acid based DESs (A) DES- 1 (ChCl + l-Methionine) (B) DES- 2 (ChCl + l- phenylalanine), (C) ChCl + l-glutamine and (D) native ChCl respectively.

The FTIR spectra of pure chloride and the DES created by their l-methionine hybrid combination are shown in Fig. 1(a). Through the FTIR characterization of the synthesized DESs, the functional groups of the DES components were investigated, and prospective structural alterations were looked into. The IR spectra reveal the presence of an alkyl group at 2850 cm^−1^ and a –OH group in vibrational bands between 3600 and 3000 cm^−1^. In the vibrational bands at 1650 and 1470 cm^−1^, respectively, primary amine and the –NH bending of the –CH_2_ group are seen, while the CN stretching is visible at 1075 cm^−1^.

Similar to this, bands 3300 and 2800 cm^−1^ in the FTIR spectrum of pure chloroform show the presence of –OH and alkyl groups, respectively. The –CN stretch at 1100 cm^−1^ was further visible in the IR spectra. In the same Fig. 6, the DES created by ChCl: L-Meth with a 1:2 M composition is also depicted. The FTIR data support the formation of DES because all of the functional groups for both ingredients were recognized in the given spectra. Hydroxyl and alkyl groups are identified by their vibrational bands at 3600 to 3000 cm^−1^ and 2850 cm^−1^, respectively. The presence of –NH and –CH_2_ groups is indicated by the bending modes at 1650 and 1470 cm^−1^. The ChCl spectra are consistent with the information found in the literature [30].

Fig. 1(a) displays the FT-IR spectrum of synthesis ChCl-l-Methionine based DESs and they exhibit Hydrogen bending at 1510.56 cm^−1^, amine (N–H) bending at 1577.22 cm^−1^, methylene (CH_2_) deformation at 1161.06 cm^−1^, amine (-NH) bend + cyanide (-CN) bend at 1410.20, 1336.73 cm^−1^, asymmetrical amine (-NH_2_) stretching at 3295.20 cm^−1^, N–C–C bending at 984.02 cm^−1^.

FTIR was conducted to investigate the formation of bonds between ChCl and L-Pheny. The FTIR spectra showed characteristic peaks of ChCl at 1352.48 cm^−1^, while the peak at 3222.21 cm^−1^ was attributed to adsorbed moisture as OH stretching. In L-Phenyl based DES a corresponding peak shift was observed at 1337.08 cm^−1^, indicating a change in wave number due to differences in electro negativity, which suggests the formation of a new bond. The shift from 1483.45 cm^−1^ to 1453.09 cm^−1^ indicates that glycerin has reacted with ChCl, and the –OH group, which includes Asymmetrical NH_2_ stretching at 3027.45 cm^−1^, Hydrogen bending at 1453.09 cm^−1^, amine (N–H) bending at 1660.71 cm^−1^, and asymmetrical amine (-NH_2_) stretching at 3027.45 cm^−1^, Wagging NH_2_ + CO out of phase, Wagging CO at 749.81 cm^−1^ which acts as a H-bond donor, has produced a bond with chloride in ChCl, as illustrated in Fig. 1(B).

The FTIR spectra of DES made with ChCl and L-Glu at a 1:2 M ratio are shown in Fig. 1(C). An ammonium quaternary salt containing the counterion chloride is known as ChCl. The vibrational bands between 2850 and 2800 cm^−1^ are where the –CH methyl groups may be seen. The bands at 1290.88 cm^−1^, meanwhile, stand in for the –CH_2_ and –CN functional groups, respectively. The vibrational bands between 3000 and 3600 were used to refer to the –OH groups, whereas 2850 was used to refer to the –CH aliphatic groups. The vibrational bands at 1573.69 and 1403.95 cm^−1^, methylene (-CH_2_) deformation at 1290.88 cm^−1^, hydrogen bending at 1573.69 cm^−1^ and 1403.95 cm^−1^, amine (N–H) bending at 1652.21 cm^−1^, and asymmetrical amine (-NH_2_) stretching at 3210.43 cm^−1^, respectively, represent the –NH, –CH_2_, and –CN functional groups, respectively. The evolution of DES and the absence of reaction species are supported by the absence of any new functional groups in the DES spectrum.

The FT-IR spectra of ChCl in Fig. 1(D), and exhibit asymmetrical –NH_2_ stretching at 3222.21 cm^−1^, N–H bending at 1672.93 cm^−1^ and 1611.62 cm^−1^, H bending at 1483.45 cm^−1^, NH bend + CN bend at 1352.48 cm-1 cm^−1^, CH_2_ deformation at 1149.07 cm^−1^, C–C stretching + other vibrations at 1090 cm^−1^

### 3.2 Determination the CMC of 1-decyl-3-methyl imidazolium chloride in the presence of three amino acids based DESs

**Fig. 2:**
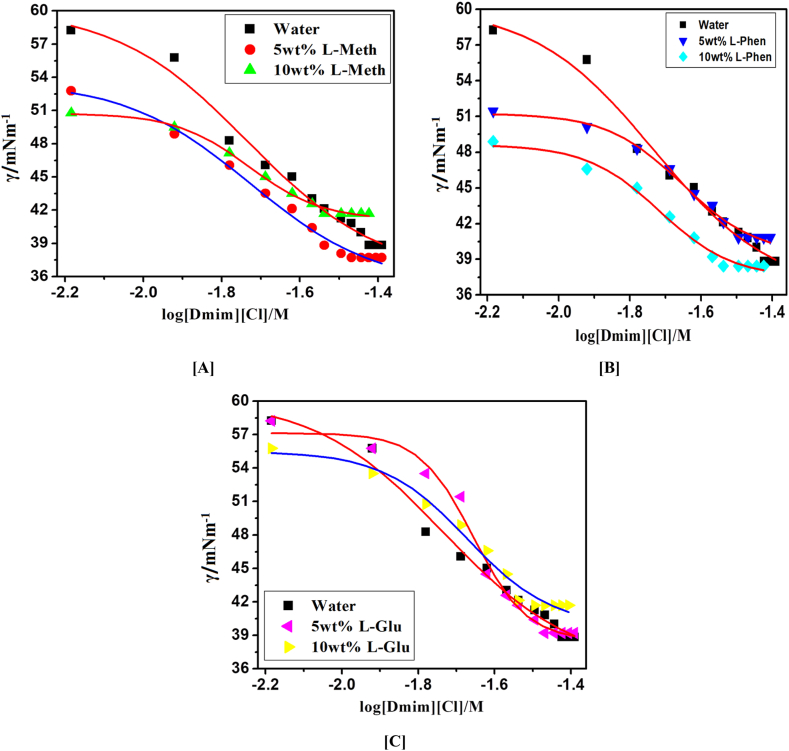
Plotted surface tension (γ) against the (log[Dmim][Cl]/M) within 5 and 10 wt% of [A] L- Metha, [B] Phen and [C] L-Glu based DESs.

**Fig. 3:**
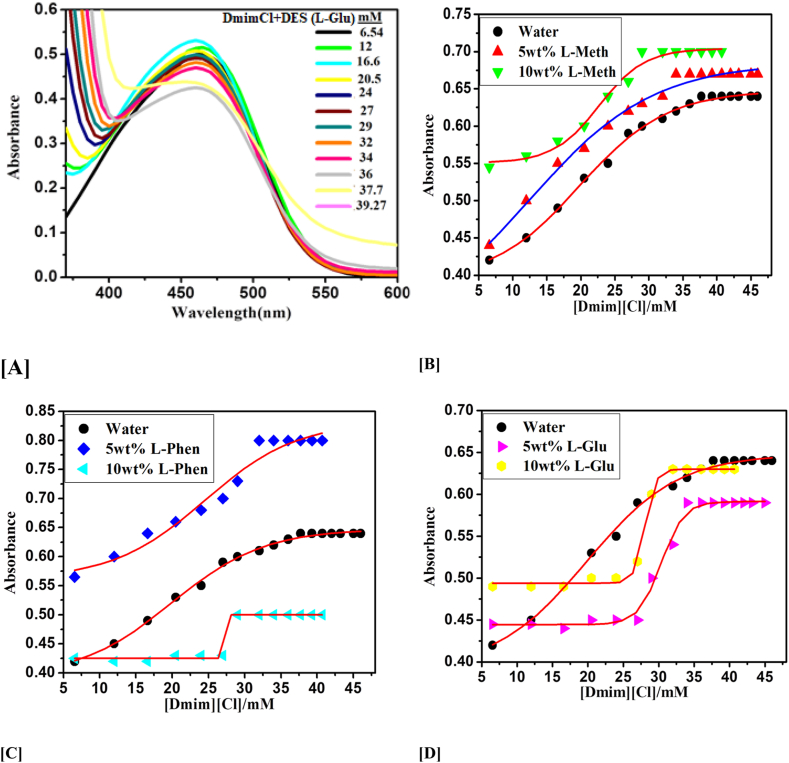
**(A)** UV–vis absorption spectra of methyl orange probe in the presence of various 1-decyl-3-methylimidazolium chloride concentrations (M). Plots the absorbance (nm) versus 1-decyl-3-methylimidazolium chloride concentration (M) in the presence of 5 and 10 wt% of [B] L- Metha, [C] Phen and [D] L-Glu based DESs.

**Fig. 4:**
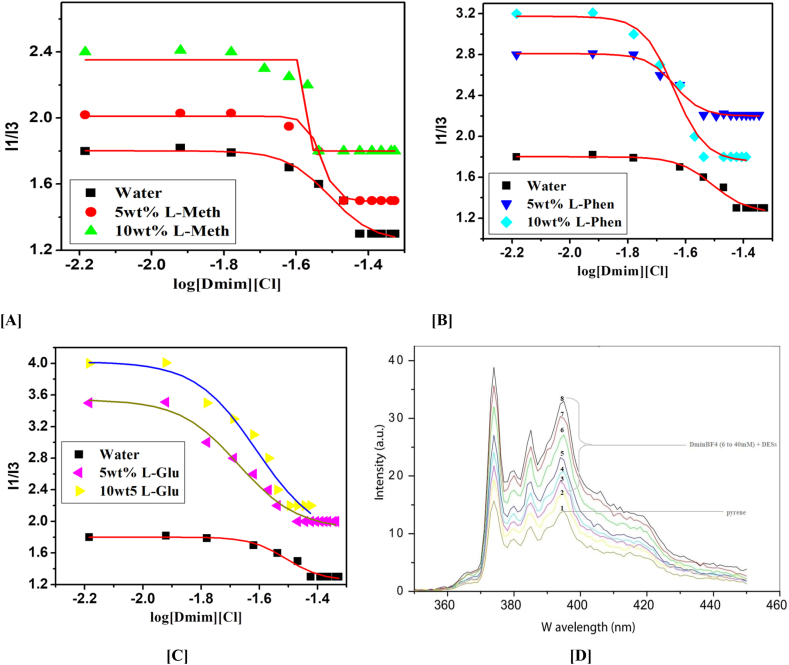
Pyrene (1 M) I1/I3 intensity ratio versus log [Dmim][Cl] (M) at ambient conditions, with 5 and 10 wt% of [A] L- Metha, [B] Phen, [C] L-Glu based DESs, and [D] fluorescence spectra of pyrene in presence and absence of DESs with variation of DmimCl concentration (6–40 mM) (max = 337 nm and slit width 2.5 nm and 1 nm).

**Scheme 4:**
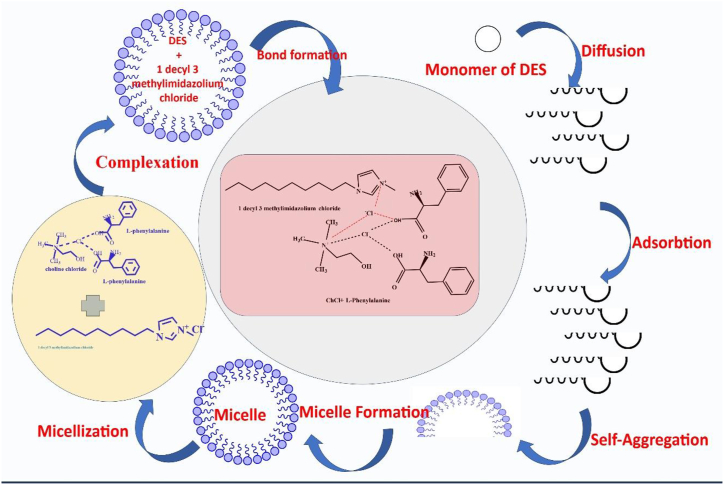
Micellization behaviour of DmimCl with DES.

We have studied the CMC values of IL DmimCl with 5 and 10 wt% of three amino acids based DESs *i.e.,* DES-1 (ChCl: l-methionine), DES-2 (ChCl: l-phenylalanine), and DES-3 (ChCl: l-glutamine) by using stalagmometric, UV–visible (methyl orange as a probe), and fluorescence (pyrene as probe) methods were used in our investigation. As a result of the fact that the experimental CMC values generated for various systems rely on the techniques, CMC has been assessed, and CMC values for all possible combinations have been given in Table 2. Fig. 2, Fig. 3, Fig. 4 clearly shows that the addition of DESs in DmimCl solutions readily reduces the CMC (38–29 mM) and micellization behavior is more rapid. Table 2 also shows that a decrease in the value of CMC occurs as 5 and 10 wt% of DESs. A significant change in the surface tension of pure aqueous solution was observed 5 and 10 wt% of DESs were added, suggesting that IL behaves as a surface-active component. The DES was associated with the DmimCl micelles, thereby reducing the CMC of the micelles. As the concentration of DES increases, the value of γ is decreased, reaches a breakpoint known as the CMC, and then remains nearly constant. All these transformations are illustrated in Table 2 and Fig. 2, Fig. 3, Fig. 4. In our previous work [31], we studied the effect of amino acids on the micellization behavior of DmimBF_4_ by stalagmometric, UV–visible (methyl orange was used as a probe), and fluorescence (pyrene as probe) methods**.** We observed the CMC values were reduced in the presence of amino acids. The micellization behavior of DmimCl with DES is shown in Scheme 4.

**Table 2:** The critical micelle concentration (CMC) of 1-decyl-3-methylimidazolium chloride was calculated using surface tension, fluorescence, and UV–vis spectroscopy techniques in the presence and absence of 5 and 10 wt% of each of the three deep eutectic solvents, l-methionine (DES1), l-phenylalanine (DES2), and l-glutamine (DES3).

DESs System	CMC (mM)		
	Surface tension	Fluorescence (Pyrene)	UV–vis (Methyl orange)
Water	38.7 ± 0.05	37 ± 0.02	36.5 ± 0.03
5 wt% ChCl-l-Methionine (DES1)	34.2 ± 0.03	33.5 ± 0.05	34.3 ± 0.04
10 wt% ChCl-l- Methionine (DES1)	29.3 ± 0.05	29.8 ± 0.03	30 ± 0.02
5 wt% ChCl-l-Phenylalnine (DES2)	32.4 ± 0.03	32 ± 0.05	32.8 ± 0.05
10 wt% ChCl-l- Phenylalnine (DES2)	29 ± 0.02	29.5 ± 0.04	29.2 ± 0.03
5 wt% ChCl-l-Glutamine (DES3)	34 ± 0.04	34.3 ± 0.02	34.5 ± 0.02
10 wt% ChCl-l-Glutamine (DES3)	31 ± 0.03	32 ± 0.05	31.5 ± 0.05

#### 3.2.1 Surface tension

The surface tension curves of DmimCl in aqueous solutions without DESs are shown in Fig. 2(A–C), and the surface tension curves of DmimCl at 5 and 10 wt% of DES-1 (ChCl: l-methionine), DES- 2 (ChCl: l-phenylalanine), and DES- 3 (ChCl: l-glutamine) are shown in Fig. 2(A–C). The estimated CMC values for the surface tension curves are displayed in Fig. 2(A–C) together with the weight percentage of extra DESs. In aqueous solutions free of any other DESs, IL DmimCl has a CMC value of 38.70 mM. The values discovered in the literature and those found by the CMC coincide fairly well.

The surface tension approach has been used to examine the adsorption behavior and surface characteristics of the aqueous mixes of DmimCl and DESs. The surface tension (γ) of water is reduced by lowering cohesive connections between the water molecules when mixes of DmimCl and DESs are added to water. Both types of amphiphilic molecules become adsorbed at the air-water interface and form mixed monolayers. The CMC of the mixture is given by the drop in value, which increases with the concentration of the mixed solution before stabilizing at a constant value as described in the preceding section.

In the presence of the three amino acid-based DESs DES- 1 (ChCl: l-methionine) (1:2), DES- 2 (ChCl: l-phenylalanine) (1:2), and DES- 3 (ChCl: l-glutamine), the CMC values of these DmimCl initially decline significantly, but thereafter decrease much more slowly or almost level off (Fig. 2(A–C)). Successfully reducing electrostatic attraction between intermolecular head groups is possible with increased weight % of DESs. The electrostatic repulsion may practically become invariable after the wt% reaches a particular size, at which time the CMC values become constant. More effective than Phen or Metha at the same weight percentage is Glu. DESs are better at encouraging the aggregation of ionic liquids than organic or inorganic salts.

The experimental surface tension error is (1 mN/m). As shown, when the experimental errors are taken into account, DES-1 (ChCl: l-methionine), DES-2 (ChCl: l-phenylalanine), and DES-3 (ChCl: l-glutamine) practically do not affect the CMC values. In other words, All DESs can greatly increase the close packing of the cationic gemini surfactant molecules at the air-water interface by significantly lowering the CMC and CMC values [25].

#### 3.2.2 UV–visible spectroscopy

Methyl orange (MO) is employed as a probe to examine the CMC of 1-decyl-3-methylimidazolium chloride (DmimCl) using contemporary UV–vis absorption spectroscopy. The presence of DmimCl causes the MO dye's largest absorption peak to undergo a bathochromic shift (red shift) between 420 and 460 nm. DESs aggregates that have developed and are presently below the CMC are the cause of pre-micellization red shifts. Frequently, the absorption peak coincides with the CMC, the sigmoid's core. Table 2 lists the estimated CMC values for 1-decyl-3-methylimidazolium chloride. UV–vis absorbance bands are shown by ionic liquid concentration (M) in Fig. 3(A). Thus, the formation of micelles is indicated by the wavelength maxima (*λ*_max_) Red Shift. As a result, DES2 at 5 and 10 wt percent decreases the CMC value of DmimCl more than other DESs [26]. Plots the absorbance (nm) versus DmimCl concentration (M) in the presence of 5 and 10 wt% of DESs was shown in Figure (B–D).

#### 3.2.3 Fluorescence

The fluorescence of pyrene can be used as a good indicator to study the self-assembly of amphiphiles in solvents by using the I1/I3 ratio. Fig. 4(D) shows the fluorescence spectra of pyrene in DmimCl/DESs solutions at different DmimCl concentrations. The DES- 1 (ChCl: l-methionine), DES- 2 (ChCl: l-phenylalanine), and DES- 3 (ChCl: l-glutamine) solutions are among them. The I1/I3 ratio of the pyrene fluorescence spectra (I1 at 374 nm and I3 at 384 nm) in DmimCl/DESs solutions at various DmimCl concentrations is shown in Fig. 4(D). The I1/I3 ratio is large because of the predominantly hydrophilic environment that surrounds pyrene in the absence of DmimCl. When DmimCl is introduced to DESs at 5 and 10 wt%, the I1/I3 ratio decreases, indicating that the microenvironment around pyrene is becoming increasingly nonpolar. As DmimCl is steadily introduced, the I1/I3 ratio then slightly changes.

The concentration at which the curve generally levels off is the CMC, which denotes that micelles are formed at this concentration. Since the CMC is 0.9 mM for the DmimCl with longer alkyl chain lengths in DES3, as seen in Fig. 4(A–C), the more hydrophobic DmimCl is believed to form micelles more easily. It can be explained by the fact that amphiphilic molecules tend to aggregate in solvents primarily as a result of the solvophobic effect. Furthermore, Fig. 4(A–C) shows that at a constant DmimCl concentration, the I1/I3 ratio for the CnmimCl/DES1 solution containing DmimCl with longer alkyl chains is lower. Pyrene molecules are anticipated to dissolve in the palisade layer after the formation of micelles, and the drop in the I1/I3 ratio suggests that the micelle core's nonpolarity and the tightness of the micellar molecular arrangement have both improved. It can be assumed that DmimCl has a more compact micellar configuration due to the longer alkyl chains.

The values for the CMC were calculated using the inflection points in Fig. 4(A–C). Similar patterns can be seen in the CMC values for the self-assembly of DmimCl in the three DES solvents; specifically, the CMC values sharply decrease as the wt% of the DESs increases from 0.99 mM to 0.65 mM. The outcome demonstrates that the general behavior of DmimCl micellar aggregation in 10 wt% DES2 is comparable to that in water, i.e., the micellization of DmimCl in DES2 is primarily driven by the solvophobic effect, similar to the micellization of amphiphilic molecules in water caused by the hydrophobic effect. Contrary to expectations, DmimCl has greater CMC values in water than in DESs [27].

The surface tension and UV–visible techniques' results are in good agreement with the calculated fluorescence results. The micellization of 1-decyl-3-methylimidazolium chloride is markedly favored by the presence of DESs, and DES2 is more effective than DES1/DES3 in this regard [28].

### 3.3 Analysis of [Dmim][Cl] and DESs interactions at air-water interface

The maximum surface excess concentration (Γ_max_), the minimum area per molecule (A_min_), and the surface pressure at CMC (π_CMC_), have been calculated for various DmimCl-DESs combinations using equations (2), (3), (4), (5), and are shown in Table 3 [29].

a) Maximum surface excess of the concentration (Γ_max_)

**Table 3:** The interfacial properties of 1-decyl-3-methylimidazolium chloride with 5 and 10 wt% DES concentrations include the surface tension at CMC (γ_CMC_), the surface pressure at CMC (π_CMC_), the efficiency of adsorption (pC_20_), the minimum surface area per molecule (A_min_), and the maximum surface excess concentration (Γ_max_). Error in data = 2 %.

DESs Systems	γ_CMC_ (mNm^−1^)	Γ_max_ (mol m^−2^) 10^6^	A_min_ (m^2^ mol^−1^) 10^20^	π_CMC_ (mN m^−1^)	_P_C_20_	CPP
Water	38.82 ± 0.02	22.96 ± 0.04	0.0723 ± 0.03	33.17 ± 0.04	1.92 ± 0.05	269.809
5 wt% ChCl-l- methionine (DES1)	37.71 ± 0.03	18.47 ± 0.03	0.089 ± 0.04	33.92 ± 0.02	2.14 ± 0.04	283.535
10 wt% ChCl-L methionine (DES1)	41.68 ± 0.04	12.32 ± 0.05	0.134 ± 0.03	29.42 ± 0.03	2.27 ± 0.04	254.838
5 wt% ChCl-l-phenylalanine (DES2)	40.82 ± 0.05	14.21 ± 0.05	0.116 ± 0.05	31.17 ± 0.04	2.18 ± 0.03	283.38
10 wt% ChCl-l-phenylalanine (DES2)	38.45 ± 0.02	13.89 ± 0.03	0.119 ± 0.04	33.55 ± 0.05	2.39 ± 0.05	280.113
5 wt% ChCl-l- glutamine (DES3)	39.21 ± 0.03	25.47 ± 0.05	0.065 ± 0.06	30.32 ± 0.02	1.91 ± 0.05	328.115
10 wt% ChCl-l- glutamine (DES3)	41.68 ± 0.05	18.67 ± 0.05	0.088 ± 0.05	27.51 ± 0.03	1.98 ± 0.03	257.967

The Gibbs adsorption isotherm was used to calculate the maximum surface excess concentration (Г_max_) by Eq (2):

(2) $$\Gamma_{\max}=\left(\frac{1}{2.303nRT}\right)\left(\frac{d\gamma }{d\;\log_{10}\;C}\right)Tp$$

where, C is the concentration of the surfactant, T is the absolute temperature, and R is the gas constant (8.314 J mol^−1^ K^−1^). Since each ionic head group has one counter ion, the value of n is assumed to be 2. The variation of Γ_max_ values of DmimCl in the addition of different types of 5 and 10 wt% of DESs solution. As a result of high surface activity observed in DESs, which gives rise to the build-up tendency to aggregate around the air-water interface of DmimCl molecules. The order of Γ_max_ values for DmimCl-DESs systems is DES3 > DES1 > DES2. 5 wt% Glutamine-based DES3 was more effectively adsorbed at the air/solution interface due to the reduction of electrostatic repulsion between head groups. When there are larger anions, this may result in a slight increase in surface excess concentrations to the other surfactants.

b) The surface pressure at CMC (π_CMC_)

The efficacy of the surface tension reduction (π_CMC_), which demonstrates the capacity to reduce the surface tension of solutions, is defined as:

(3) $$\pi_{\text{CMC}}=\gamma_{0}\;-\;\gamma_{\text{CMC}}$$

where, γ_CMC_ denotes the solutions' surface tension at CMC and ϒ_0_ denotes the surface tension of distilled water. Where, γ_CMC_ is the surface tension at CMC, γ_0_ is the surface tension of pure water. Different interactions were contributed by the systems of surfactants for the effective adsorption of 5 and 10 wt% of DESs solution at the air-water interface. The maximum values of π_CMC_ have indicated more effective adsorption at the DmimCl interface due to the polar-non-polar repulsion of the 5 wt% DES1 system being significantly higher than the DES2 and DES3 system repulsions. In DES1 molecules have present the -S- group is significantly importance except other DESs. Therefore, due to the larger hydrophilic head part, more repulsion was displayed at the interface. The resulting order of π_CMC_ is DES1 > DES2 > DES3.

c) The efficiency of adsorption (pC_20_)

The following equation (4) is used to determine the adsorption efficiency (pC_20_):

(4) $$pC_{20}=-\log_{10}C_{20}$$

The concentration of the ILs surfactants, which lower the surface tension of the pure solvent by 20 mN/m, is represented by the negative log, pC_20_, in this equation. The efficiency of amphiphilic molecules for adsorption at the air/water interface is normally determined by the bulk concentration of these molecules, which causes a surface tension reduction was observed at 20 mNm^−1^ (pC_20_) from the pure solvent. Their negative logarithm of C_20_ (-logC_20_) is called pC_20_ and having a higher values indicates a larger adsorption efficiency. Due to its higher hydrophobicity and pure DmimCl has a higher pC_20_ value except a mixture of DESs was seen in Table 3. Higher pC_20_ values have shown that the combinations of DmimCl and DESs are more surface active than their solo components.

d) A minimum surface area per molecule (A_min_)

The adsorbed surfactant molecule's degree of packing and orientation is revealed by the minimum surface area per molecule (A_min_). Applying the Gibbs adsorption isotherm, the minimum area per molecules (A_min_) occupied by a single amphiphilic molecule at the air-water interface was computed.

(5) $${Α}_{\min}=\left(\frac{1}{\Gamma_{\max}}\right)N_{A}$$

The minimum area per molecule is inversely proportional to the maximum surface excess concentration so, the higher Γ_max_ value lowers comparison to A_min_ value. The order found for A_min_ is: DES2 > DES1 > DES3. This indicates that the molecules are less tightly packed for flexibility at the air-water contact. The A_min_ values increase with an increase in the 5 and 10 wt% of DESs for DmimCl. It could be demonstrated that the effect of DESs reduced the surface area of DmimCl molecule accessible.

e) The critical packing parameter (CPP) of DmimCl with DESs

The critical packing parameter (CP*P*) of DmimCl with 5 and 10 wt% of DESs solution was calculated by using the following Eqs. (6), (7), (8).

(6) $$P=\frac{V_{0}}{A_{\min}l_{c}}$$

(7) $$V_{0}=\left(27.4+26.9C_{n}\right){Å}^{3}\;\text{per}\;\text{hydrocarbon}\;\text{chain}$$

(8) $$l_{c}=\left(1.5+1.265C_{n}\right)Å\;\text{per}\;\text{hydrocarbon}\;\text{chain}$$

The value of *P* is ≤ 0.33 for the shape of a spherical micelle. The mixed system of DmimCl-DESs shows that the shape of the aggregate system changes from globule to spherical with increasing concentration. The order of CPP values for DmimCl-DESs systems is: DES3 > DES1 > DES2 and data were reported in Table 3.

### 3.4 Viscosity study

**Fig. 5:**
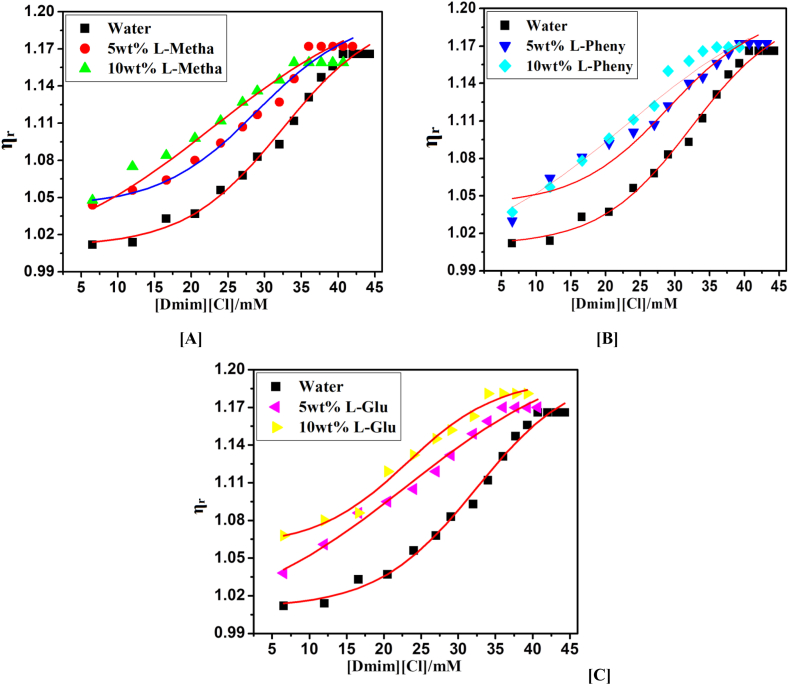
Graph plot between relative viscosity (η_r_) versus 1-decyl-3-methylimidazolium chloride (M) with 5 and 10 wt% of [A] L- Metha, [B] Phen and [C] L-Glu based DESs.

The viscosity of [Dmim][Cl] is a crucial component of their liquid characteristic and directly influences their ability to transfer charges. Conductivity, diffusion coefficient, charge transfer rate, and other transport parameters of IL [Dmim][Cl] can all be intimately affected by variations in viscosity. At a temperature of 298 K, the viscosities of four IL and a binary mixture of DESs were measured and compiled in Fig. 5(A–C). When the DES2 anion and 1-decyl-3-methylimidazolium cation combined it viscosity was observed at 8.820 cP at 298 K is produced.

**Fig. 6:**
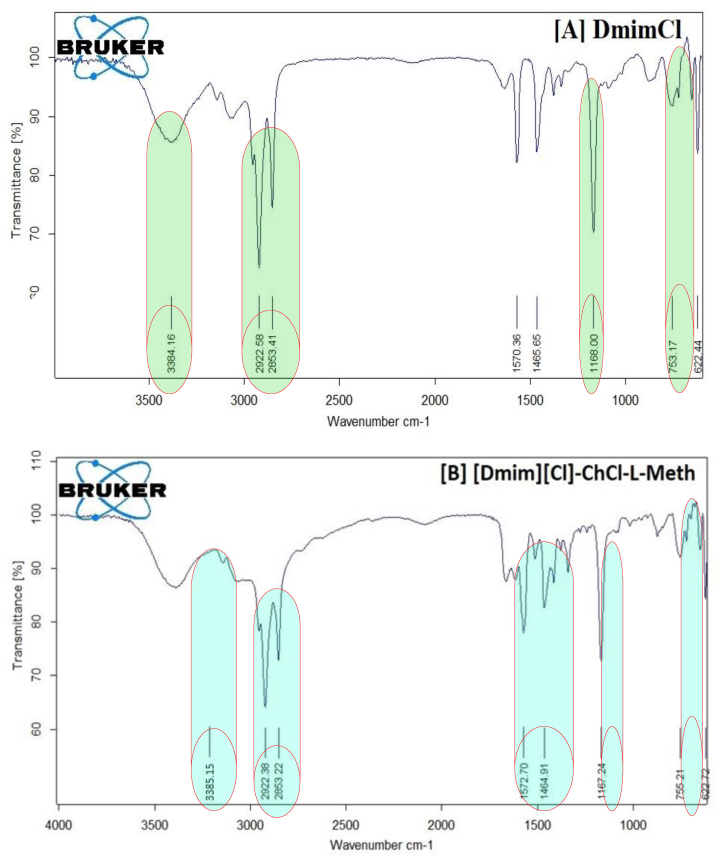

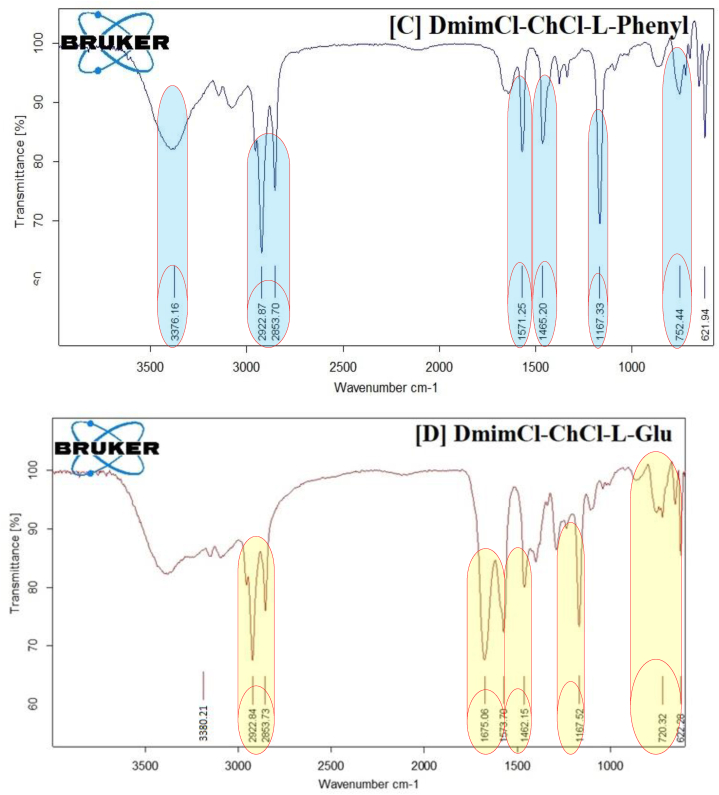
FT-IR spectra of [A] native 1 decyl 3 methyl imidazolium chloride, [B] 1 decyl 3 methyl imidazolium chloride -DES1, [C] 1 decyl 3 methyl imidazolium chloride -DES2 and [D] 1 decyl 3 methyl imidazolium chloride -DES3.

The relative viscosity of [Dmim][Cl] is 15.935 and (i) binary mixture with a 5 and 10 wt% DES1 is η_r_ = 12.57, 10.54 cP, (ii) binary mixture with a 5 and 10 wt% DES2 is η_r_ = 11.405, 8.82 cP, (iii) binary mixture with a 5 and 10 wt% DES3 is η_r_ = 12.91, 12.10 cP.

In contrast, pure IL [Dmim][Cl] has a higher relative viscosity value except DESs mixture. The DESs salts with imidazolium cations are less viscous. It has been noted that asymmetric N-substituted imidazolium cations are appropriate for the creation of low-viscous. In these circumstances, the charge delocalization and planarity's synergistic action results in low viscous IL. The lower viscosity of IL could result from less hydrogen bonding. However, compared to [IL [Dmim][Cl], L-Pheny IL [Dmim][Cl] with active hydrogen on C(2) demonstrates lesser viscosity. IL [Dmim][Cl]-Phenyl is thus more viscous than IL [Dmim][Cl]-DES1/DES3. As a result, imidazolium phenyl ILs have less viscosity than DES1/DES3 IL. Therefore, the cationic structures may have an impact on the viscosity of ILs through the interaction of charge distribution, hydrogen bonds, and van der Waals attractive force. The graph in Fig. 5(A–C) below displays the concentrations vs. the relative viscosities of various systems [30].

### 3.5 3.3 Effect of amino acid based DESs on micellization of [Dmim][Cl]

The impact of additives such as DESs on the CMC value of [Dmim][Cl] is thus crucial to be studied in both theory and practice. Table 2 demonstrates that the DESs have the same pattern on the CMC value of [Dmim][Cl]. When DESs were addition in IL[Dmim][Cl] aqueous solution, the CMC values are regularly decreased. The impact of DESs in [Dmim][Cl] aqueous solution were confirm by surface tension, UV–Vis, and fluorescence spectroscopy was shown in Fig. 2, Fig. 3, Fig. 4. In general, the attractive forces brought on by the need to minimize the exposure of the hydrophobic core to water and the repulsive head group interactions regulate the formation of [Dmim][Cl] aggregations. As a result, the CMC values of [Dmim][Cl]'s may decrease as a result of the counter ions' (Cl-). Additionally, it was discovered that the impact of cations on the CMC value was negligible.

The CMC value of [Dmim][Cl] typically tends to gradually decrease upon the introduction of the AAs based DESs L-Metha, L-Phenl, and L-Glu. Generally speaking, the polar organic molecule affects the characteristics of IL by maintaining or dissolving the water structure surrounding the hydrophobic chains [32]. l-methionine is mostly soluble in water through micelle aggregates made of IL molecules, which have an –S atom in the structure surrounding their hydrophobic chains and hence have a reduced propensity to micellize. As a result, the CMC value tends to fall a little.

**Scheme 5:**
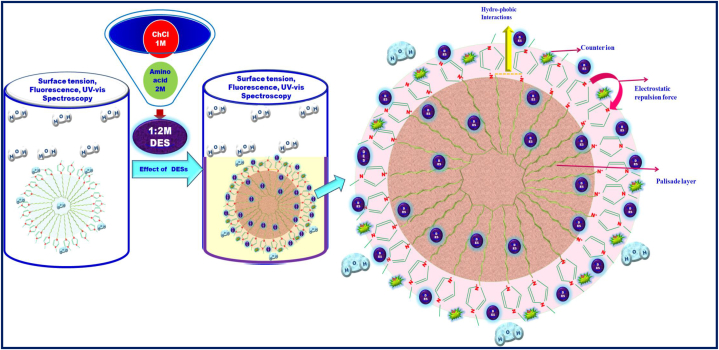
Illustration in a schematic of a [Cnmpy][Br][Dmim][Cl] micelle in water with DESs.

However, different DESs were utilized in this work to gradually reduce CMC values for two reasons: (i) AAs become less soluble in water, and an “iceberg structure” forms around the AA molecules. (ii) DES can insert into the palisade layer, which lessens the electrostatic repulsion forces between the ionic head groups in the micelle. The analysis of above results and literature findings [33] were used to represent and display the probable micelle structure in the presence of inorganic salts and organic alcohols in water in Scheme 5.

### 3.6 FTIR study of 1-decyl-3-methylimidazolium chloride and it micellar complexes in DESs

The infrared spectra, which range from 400 to 4000 cm-1 and are depicted in Fig. 6(A–D), reveal the mole fractions of DES1, DES2, and DES3 in these binary combinations.

a) IR spectra of native [Dmim][Cl]

The FTIR of [Dmim][Cl] was shown in Fig. 6(A). The existence of the numerous functional groups and the specific structure of the [Dmim][Cl] are both shown in Scheme 1. The most accurate and advanced way of investigating functional groups is the FT-IR technique. Because pure IL is hygroscopic, the O–H stretching bands, which are mostly caused by the absorbed water, may be visible in the FTIR spectrum of pure IL at a wavelength of 3384.16 cm^−1^. The [Dmim][Cl] structure exhibits the long chain symmetric and asymmetric stretching C–H vibration at a frequency of 2922.58 cm^−1^. The aromatic methylene C–H bending IR frequency was observed at 753.17 cm^−1^ for the imidazolium ring, while the aromatic C=C bending IR frequency was observed at 1570.36 cm^−1^. The C–N function group is present in the chemical structure of IL, and this stretching frequency was measured at 1465.65 cm^−1^. A lengthy C-chain contains the [Dmim][Cl] is IF frequency measured at 1168.00 cm^−1^, which indicates the presence of C–H in-plane bending [34]. Table 4 shows the FTIR data of 1-decyl-3-methyl imidazolium chloride and its mixture with DESs based on amino acids have distinct infrared absorption bands.

b) IR spectra of 1-decyl-3-methyl imidazolium chloride – DES1

**Table 4:** 1 Decyl 3 methyl imidazolium chloride and its mixture with DESs based on amino acids have distinct infrared absorption bands. Error in data = 2 %.

Functional Group	Wavenumber (cm^−1^)			
	[Dmim][Cl]	[Dmim][Cl]-ChCl-L-Meth	[Dmim][Cl]-ChCl-L-Glu	[Dmim][Cl]-ChCl-L-Phenyl
stretching the v[O–H] vibration band	3384.16	3385.15	3380.21	3376.16
Alkyl chains of v[C–H] stretching vibrating symmetrically and asymmetrically moiety	2922.58, 2853.41	2922.38, 2853.22	2922.10, 2851.25	2921.05, 2853.25
C–H in-plane-bending	1168.00	1167.24	1169.70	1169.11
Chain of methylene (rocking mode)	753.17	755.21	753.11	755.15
	1465.65	1466.68	1466.70	1466.50
Aromatic C=C Bending	1570.36	1572.70	1572.80	1572.85

FTIR is used to investigate the complex formation between [Dmim][Cl] and DES1 as shown in Fig. 6(B). A long chain called [Dmim][Cl]IL that is present in the symmetric and asymmetric stretching CH2 vibration of the alkyl chains function group exhibits an initial peak at 2922.58 cm^−1^ and is moved to 2922.38 cm^−1^, causing a modest change in IR frequency of 0.20 cm^−1^ to be seen. These results demonstrate an additional IR frequency shifting of 2.34 cm^−1^. This [Dmim][Cl] presence of an imidazolium ring presents an alternate aromatic C=C bending in IR frequency recorded at 1570.36 cm^−1^ and is shifted to 1572.70 cm^−1^. These results demonstrate the minute changes in IR frequency at 1.24 cm^−1^ caused by the existence of long chains in the IL functional group C–H in-plane bending at 1168.0 cm^−1^ and 1167.24 cm^−1^. Small variations in IR frequency of 2.02 cm^−1^ are seen as a result of the shift in the aromatic C–H bending from 753.17 cm^−1^ to 755.21 cm^−1^ [35].

c) IR spectra of 1-decyl-3-methyl imidazolium chloride – DES2

FTIR is used to investigate the complex formation between [Dmim][Cl] and DES2 as shown in Fig. 6(C). A long chain called [Dmim][Cl]IL that is present in the symmetric and asymmetric stretching CH2 vibration of the alkyl chains function group exhibits an initial peak at 2922.58 cm^−1^ and is moved to 2922.10 cm-1, causing a modest change in IR frequency of 0.48 cm^−1^ to be seen. These results demonstrate a further IR frequency shifting of 2.44 cm^−1^. This [Dmim][Cl] presence of an imidazolium ring presents an alternate aromatic C=C bending in IR frequency recorded at 1570.36 cm^−1^ and is shifted to 1572.80 cm^−1^. Long chain presence in IL is functional group C–H in-plane bending exhibited the IR frequency at 1168.0 cm^−1^ and is moved to 1169.70 cm^−1^; these results demonstrate the slight variations in IR frequency at 1.70 cm^−1^. Small changes in IR frequency of 0.06 cm^−1^ are seen as a result of the shift in the aromatic C–H bending from 753.17 cm^−1^ to 753.11 cm^−1^.

d) IR spectra of 1-decyl-3-methylimidazolium chloride-DES3

FTIR is used to investigate the complex formation between [Dmim][Cl] and DES3 as shown in Fig. 6(D). The first peak of the [Dmim][Cl]IL long chain, which is present in symmetric and asymmetric stretching CH2 vibration of the alkyl chains function group, is displayed at 2922.58 cm^−1^ and is displaced to 2921.05 cm^−1^. This causes a minor change in IR frequency of 1.53 cm^−1^ to be noticed. These results demonstrate a further IR frequency shifting of 2.41 cm^−1^. This [Dmim][Cl] presence of an imidazolium ring presents an alternate aromatic C=C bending in IR frequency recorded at 1570.36 cm^−1^ and is shifted to 1572.85 cm^−1^. Long chain presence in IL is functional group C–H in-plane bending exhibited the IR frequency at 1168.0 cm^−1^ and is moved to 1169.11 cm^−1^; these results demonstrate the slight variations in IR frequency at 1.11 cm^−1^. Small variations in IR frequency of 1.98 cm-1 are seen as a result of the shift in the aromatic C–H bending from 753.17 cm^−1^ to 755.15 cm^−1^ [36].

According to the shift found, the C4–H in the IL and the solvent of PYR may produce the weakest hydrogen bonds. With the other DES solvents, the C–H formations produced from IL do not show any distinguishable variations. Due to the zero value of the solvent acidity, the four solvents cannot also act as hydrogen-bond donors, making it less likely that they will obstruct interactions between the DESs and the imidazolium protons [37].

## 4 Conclusions

In the current work, surface tension, fluorescence and UV–vis absorption spectra were used to analyze the micellization behavior and surface activity of IL [Dmim][Cl] within amino acid-based DESs aqueous solution. The better surface characteristics of IL [Dmim][Cl] over conventional surfactants with the same alkyl chain length are shown [38]. It was demonstrated that the presence of AAs-based DESs had a substantial impact on the micellization behavior of [Dmim][Cl] in aqueous solutions. The CMC values of all systems decreased significantly in the presence of all the studied DESs. DES2 is the most effective at promoting IL aggregation, as compared to DES1 and DES3. Different types of interactions such as, electrostatic, H-bonding and hydrophobic interactions results in promoting the aggregation of IL [Dmim][Cl] within amino acid-based DESs solutions [39]. Vesicles and micelles coexist in [Dmim][Cl] solution, a salt-free solution, whereas only micelles do so in [Dmim]solution. It has been shown that a tiny amount of solvent can significantly reduce the viscosity of pure IL, with DESs having the biggest effects [40]. According to the FTIR spectral shift, the C4–H in the IL and the solvent of DES may provide the weakest hydrogen bonding. There are no obvious distinctions between the C–H hydrogen-bonding structures in DESs and the other solvents. Earlier, surface tension, conductivity, UV–Vis, fluorescence, FT-IR, and NMR methods have all been used to synthesize, characterize, and study the effect of DESs on the micellization behavior of ionic liquids and conventional surfactants [41,42]. This observation is in good agreement with our findings.

## CRediT authorship contribution statement

**Manoj Kumar Banjare:** Writing – review & editing, Writing – original draft, Visualization, Validation, Supervision, Software, Resources, Project administration, Methodology, Investigation, Funding acquisition, Formal analysis, Data curation, Conceptualization. **Benvikram Barman:** Methodology, Investigation, Formal analysis, Data curation. **Kamalakanta Behera:** Writing – review & editing, Writing – original draft, Validation, Supervision, Software, Resources, Methodology, Funding acquisition, Conceptualization. **Javed Masood Khan:** Writing – review & editing, Software, Project administration, Funding acquisition, Formal analysis, Conceptualization. **Ramesh Kumar Banjare:** Writing – review & editing, Visualization, Software, Methodology, Formal analysis, Data curation. **Siddharth Pandey:** Writing – review & editing, Writing – original draft, Visualization, Supervision, Resources, Funding acquisition, Formal analysis, Data curation, Conceptualization. **Kallol Kumar Ghosh:** Writing – review & editing, Writing – original draft, Visualization, Supervision, Methodology, Investigation, Funding acquisition, Formal analysis, Data curation, Conceptualization.

## Declaration of competing interest

The authors declare the following financial interests/personal relationships which may be considered as potential competing interests:DR. MANOJ KUMAR BANJARE reports financial support was provided by 10.13039/501100002383King Saud University. DR. MANOJ KUMAR BANJARE reports a relationship with NA that includes: non-financial support. DR. MANOJ KUMAR BANJARE has patent NA pending to NA. NA If there are other authors, they declare that they have no known competing financial interests or personal relationships that could have appeared to influence the work reported in this paper.
